# High Levels of Outpatient Antibiotic Prescription at a District Hospital in Ghana: Results of a Cross Sectional Study

**DOI:** 10.3390/ijerph191610286

**Published:** 2022-08-18

**Authors:** Obed Kwabena Offe Amponsah, Sharath Burugina Nagaraja, Nana Kwame Ayisi-Boateng, Divya Nair, Karlos Muradyan, Phanuel Seli Asense, Osei Kwaku Wusu-Ansah, Robert Fraser Terry, Mohammed Khogali, Kwame Ohene Buabeng

**Affiliations:** 1 Department of Pharmacy Practice, Kwame Nkrumah University of Science and Technology, Kumasi 00233, Ghana; 2 Department of Community Medicine, ESIC Medical College and PGIMSR, Bengaluru 560010, India; 3 Department of Medicine, Kwame Nkrumah University of Science and Technology, Kumasi 00233, Ghana; 4 University Hospital, Kwame Nkrumah University of Science and Technology, Kumasi 00233, Ghana; 5 International Union against TB and Lung Disease (The Union), 75006 Paris, France; 6 Tuberculosis Research and Prevention Center, Yerevan 0014, Armenia; 7 Special Programme for Research and Training in Tropical Diseases (TDR), World Health Organisation, 1211 Geneva, Switzerland

**Keywords:** antibiotic prescription, outpatients, *AWaRe* classification, Ghana, SORT IT, antimicrobial stewardship, electronic medical records, operational research, antimicrobial resistance

## Abstract

Background: Monitoring of antibiotic prescription practices in hospitals is essential to assess and facilitate appropriate use. This is relevant to halt the progression of antimicrobial resistance. Methods: Assessment of antibiotic prescribing patterns and completeness of antibiotic prescriptions among out-patients in 2021 was conducted at the University Hospital of Kwame Nkrumah University of Science and Technology in the Ashanti region of Ghana. We reviewed electronic medical records (EMR) of 49,660 patients who had 110,280 encounters in the year. Results: The patient encounters yielded 350,149 prescriptions. Every month, 33–36% of patient encounters resulted in antibiotic prescription, higher than the World Health Organization’s (WHO) recommended optimum of 27%. Almost half of the antibiotics prescribed belonged to WHO’s *Watch* group. Amoxicillin–clavulanic acid (50%), azithromycin (29%), ciprofloxacin (28%), metronidazole (21%), and cefuroxime (20%) were the most prescribed antibiotics. Antibiotic prescribing parameters (indication, name of drug, duration, dose, route, and frequency) were documented in almost all prescriptions. Conclusions: Extending antimicrobial stewardship to the out-patient settings by developing standard treatment guidelines, an out-patient specific drug formulary, and antibiograms can promote rational antibiotic use at the hospital. The EMR system of the hospital is a valuable tool for monitoring prescriptions that can be leveraged for future audits.

## 1. Introduction

Antibiotics are life-saving drugs commonly used in clinical practice worldwide [1,2]. However, their irrational and rampant use has contributed to the emergence and spread of antimicrobial resistance (AMR), which is one of the greatest threats to human health [3]. AMR negatively impacts health outcomes and has substantial financial and societal implications, which disproportionately affect low- and middle-income countries (LMICs) [4,5,6,7,8].

According to the World Health Organization (WHO), the rate of antibiotic prescribing in general practice should be less than 27% of all prescriptions [9,10,11]. However, this threshold has been exceeded in different settings across the globe [11,12,13]. To address irrational use of antibiotics, the WHO launched a global action plan (GAP) on AMR in 2015. One of the main pillars of the GAP is to optimize the use of antimicrobials, including antibiotics [14]. In this regard, WHO has categorized antibiotics as *AWaRe*—Access, Watch, and Reserve [15]. The Access category includes first and second line antibiotics for empirical treatment needed for common infections, and these should be available in all health care settings. The Watch category includes antibiotics with higher potential to develop resistance and their use as first and second choice treatment should be limited, while the Reserve category is restricted as “last resort” antibiotics whose use should be reserved for special situations with multidrug-resistant bacterial infections where alternative treatments have failed [15,16]. Countries in Africa are projected to carry one of the greatest burdens of AMR, with over 4 million AMR-attributable deaths each year [17]. Therefore, monitoring antibiotic prescription patterns is one of the recommended strategies to prevent their overuse [18,19]. Such audits require proper documentation of the prescribed antibiotics in terms of indication, name, doses, route, and frequency of administration [20]. 

In 2017, Ghana developed a national action plan to combat AMR based on the principles of WHO GAP [14,21]. Several studies have reported on the rate of antibiotic prescribing in different countries including Ghana in both inpatient and outpatient settings ranging from 34% to 82% [22,23,24,25,26,27]. However, the studies that were conducted in Ghana focused on specific regions and only provided snapshots of the rate of antibiotic prescribing in outpatient settings of those regions [28,29,30]. There are no studies conducted among outpatients in the Ashanti region of Ghana where a stewardship programme among inpatients is under implementation in the University Hospital of Kwame Nkrumah University of Science and Technology (KNUST). In addition, no studies from Ghana assessed the completeness of documentation of the prescribed antibiotics in outpatient settings. This could serve as important baseline information on antibiotic use for the stewardship programme in the University Hospital, KNUST. Additionally, it will serve as a yardstick for the optimization of antibiotic use and future planning. 

We conducted a study on antibiotic prescribing patterns and completeness of documentation among out-patients of the University Hospital, KNUST, in the Ashanti Region of Ghana in 2021. The specific objectives were to assess (a) demographic and clinical characteristics of patients who received antibiotics, (b) the monthly trend of antibiotic prescribing per WHO *AWaRe* classification, and (c) completeness of antibiotic prescriptions.

## 2. Materials and Methods

### 2.1. Study Design

This was a cross-sectional study using routinely collected electronic data from outpatient medical records of the University Hospital, KNUST.

### 2.2. Settings

#### General Setting

Ghana is a West African country with a population size of 30.8 million as of 2021. Administratively, the country is divided into 16 regions with Accra being the capital city [31]. The health care services in Ghana are mainly organized through a three-tiered system (primary, secondary, and tertiary). There are 1812 public hospitals, 1356 private hospitals, and 204 mission hospitals (owned by religious organizations) in Ghana [32]. The health services in the country are provided predominantly by public (government) hospitals. Some of these public hospitals are classified as quasi-governmental hospitals because they are funded by both the government and private sector. Antibiotics are widely available in both public and private health sectors and also in pharmacies across the country. Over-the-counter antibiotics are also available and accessible even without prescriptions [33]. Some antibiotics are provided to patients free of cost through the National Health Insurance Scheme (NHIS) and private health insurance while others have to be purchased out-of-pocket [34].

### 2.3. Specific Settings

The University Hospital, KNUST, is a quasi-government district-level hospital with 135 beds and has patient footfall of at least 100 patients a day at the out-patient department (OPD). The hospital is located in the second largest administrative region, the Ashanti Region [31]. The hospital serves the university community and a catchment area of 303,016 people in the Oforikrom Municipality as well as its environs [35]. The services provided at the University Hospital include care in internal medicine, family medicine, surgery, paediatrics, obstetrics and gynaecology, dental care, mental health, infectious diseases, emergency services, urology, haematology, otorhinolaryngology, ophthalmology, and neurology, among others. Many peripheral health facilities in the region refer patients to this hospital for specialist care. The hospital serves as a teaching facility for students in the health sciences across the country.

#### 2.3.1. Outpatient Department of the University Hospital, KNUST

The OPD of the University Hospital is the first port of call for patients presenting to the hospital. When a patient visits the hospital for the first time, an account is created for them by the Records Department and a unique identification number is assigned. During subsequent visits, patients go through a data clerk from the Records Department who retrieves their account from the electronic medical record (EMR). Following this, the patient is evaluated by a physician/physician assistant and the relevant clinical findings are updated on the EMR. 

#### 2.3.2. Process for Antibiotic Prescription and Dispensing in OPD of University Hospital, KNUST

Following consultation at the OPD, the patient’s prescription is entered by the physician/physician assistant directly into the EMR. Information on the prescribed treatment is, thus, transferred electronically to the pharmacy department for dispensing to the patient. In case the prescribed antibiotics are not available at the hospital pharmacy, a paper-based prescription is generated at the pharmacy for the patient to purchase from outside the hospital.

#### 2.3.3. Outpatient Medical Records

For documentation and maintenance of records and reports, the hospital relies on the EMR system developed in-house connecting the various departments of the hospital. It is largely a local area network (LAN)-based system with some components being web-based. Demographic information such as age and sex of patients attending the outpatient department (OPD) are recorded in the EMR at the time of first visit to the hospital. Clinical information pertaining to the patient is updated on the EMR by the physician/physician assistant at the time of consultation. This includes medical history, comorbidities, diagnosis, as well as the prescribed treatment.

### 2.4. Study Population

The study included prescriptions of all patients treated at the OPD of the University Hospital between January and December 2021.

### 2.5. Data Variables and Analysis

Data on demographic and clinical characteristics and drug prescriptions of patients was retrieved from the EMR system of the OPD.

Data from the EMR was retrieved in a MS Excel CSV format, cleaned, and processed using Python in Jupyter Notebook, a web-based interactive computing platform [36], and analyzed using STATA^®^ (version 16.0 Copyright 1985–2019 StataCorp LLC, College Station, TX, USA). Antibiotics prescribed were categorised according to the WHO *AWaRe* classification into Access, Watch, or Reserve group. A patient was considered to have received an antibiotic if any of the prescriptions retrieved against their unique identifier contained an antibiotic. A patient encounter referred to a consultation on the same day in a department of the OPD. Each medicine prescribed during a patient encounter was considered as a separate prescription. Frequencies and proportions of prescriptions of antibiotics were analysed with different units of analysis: patient encounters and prescriptions. Completeness of antibiotic prescriptions was assessed in terms of six quality indicators: (1) indication for treatment, (2) name of antibiotic prescribed, (3) duration, (4) dose of antibiotic prescribed, (5) route of administration, and (6) frequency of administration. 

## 3. Results

Between January and December 2021, 49,660 patients had visited the OPD of the KNUST Hospital resulting in 110,280 patient encounters. Data review and cleaning yielded 350,149 prescriptions that were analyzed further.

### 3.1. Patient Demographics and Clinical Characteristics

More than half of the patients were male (53.9%) with over 45% being between the ages of 15 and 24 years. The mean age was 29 ± 18 years and ranged between 0 to 105 years. Of all those with recorded chronic conditions (5242), 68.4% had hypertension which was the most common (Table 1).

### 3.2. Antibiotic Use Based on Age, Sex and Clinic

A higher proportion of patient encounters involving children under 15 years more often included prescribed antibiotics (54.5%) compared to the older age groups. Antibiotics were uniformly prescribed in encounters with females (35%) and males (36%). The majority of encounters (54%) in the dental clinic more often had prescribed antibiotics (Table 2).

### 3.3. Monthly Trends in Antibiotic Prescriptions

**Figure 1:**
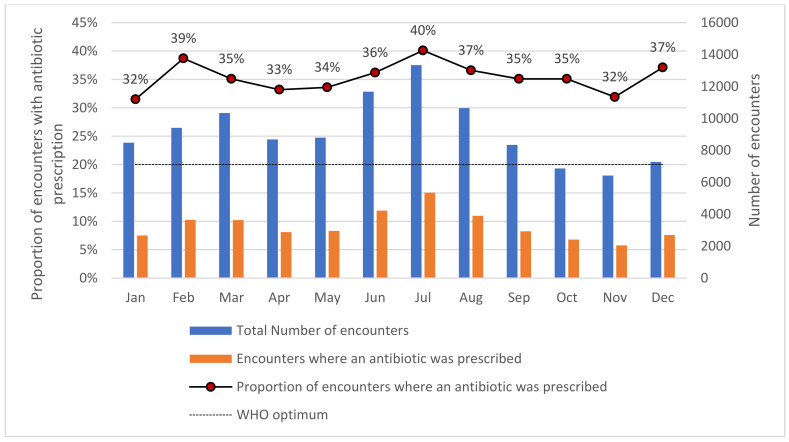
Monthly trend of antibiotic prescriptions in the outpatient department of University Hospital, KNUST, in Ghana between January and December 2021, KNUST—Kwame Nkrumah University of Science and Technology.

During the study period, 110,280 patient encounters occurred in the OPD, out of which 39,339 (36%) resulted in the prescription of an antibiotic. The trends of antibiotic prescriptions varied from 32–40% during the year 2021. The hospital found increased prescriptions in the months of February, July, and December. Across all months, the percentage of encounters with antibiotics prescribed exceeded the desired optimal value of 20% as recommended and in line with WHO prescribing indicators (Figure 1).

### 3.4. Monthly Antibiotic Prescriptions According to the WHO AWaRe Classification

**Figure 2:**
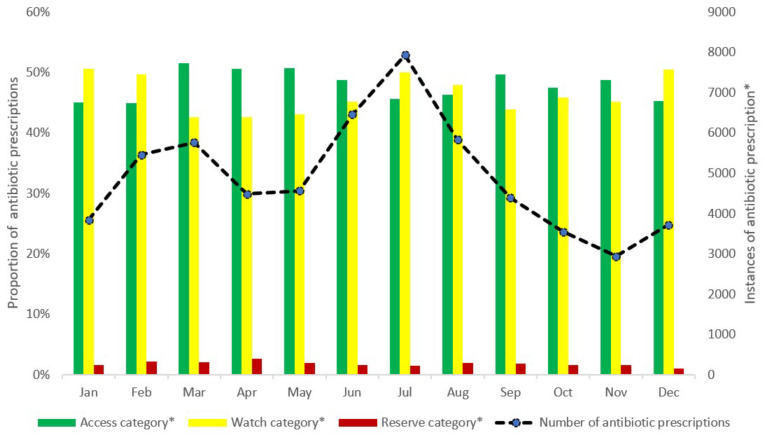
Monthly antibiotic prescriptions according to the WHO *AWaRe* (Access, Watch, Reserve) classification among patients visiting the outpatient department of University Hospital, KNUST, in Ghana in 2021. * Proportions for each of the *AWaRe* classification of antibiotics calculated with total number of antibiotic prescriptions as denominator. KNUST—Kwame Nkrumah University of Science and Technology.

In 2021, there were 110,280 patient encounters with 350,149 drug prescriptions. The percentage of antibiotic prescriptions out of all prescriptions in the year was 17% (58,885 out of 350,149) with a monthly range between 15–18%. Antibiotics belonging to the *Access* category contributed to 45–52% of all antibiotic prescriptions every month. Similarly, antibiotics belonging to the *Watch* category contributed to 43–51% of all antibiotic prescriptions every month. The prescription of *Reserve* antibiotics was found to be minimal, contributing to less than 3% of all antibiotic prescriptions every month (Figure 2).

### 3.5. Pattern of Antibiotic Prescriptions

Among the Access group of antibiotics, amoxicillin–clavulanic acid was the most commonly (50%) prescribed followed by metronidazole (21%). Among the Watch group of antibiotics, azithromycin (29%) and ciprofloxacin (28%) were the most commonly prescribed (Table 3).

### 3.6. Prescribing Indicators

All antibiotic prescribing parameters (indication, name, doses, route of administration, duration, and frequency of administration of antibiotics) were documented in all 58,885 prescriptions with the exception of 44 prescriptions where the names of a few specific antibiotics were not documented. The documented names of antibiotics were not uniform in the hospital; there were discrepancies in terms of their spellings and the marketing brands to which they belonged. Though indications were captured, they did not follow the standard classification system.

## 4. Discussion

To our knowledge, this is the first study that assessed outpatient antibiotic prescribing practices from a district hospital in the Ashanti region of Ghana. Our study findings reveal that more than a third of patient encounters in the OPD had antibiotics. There was a low prescription of WHO *Reserve* antibiotics across the year. The practice of completing documentation of prescription parameters was followed in almost all antibiotic prescriptions. In the following paragraphs, we discuss the implications of our findings.

(a) Antibiotic prescriptions: Of all patient encounters in the study, antibiotics were prescribed in over 36% of encounters throughout the year, which is higher than the WHO recommended levels of 27% of encounters [11]. However, this is still lower than findings from similar studies which reported prescription levels of 46% from Bangladesh [37], 60% from Ghana [38], and 79% from Pakistan [39]. There was no significant monthly or seasonal variation in antibiotic prescribing across the year. Though the month of July witnessed higher antibiotic prescription rates, this could be attributed to the increased patient footfall in that period when the COVID-19 pandemic was at its peak in Ghana [40,41,42]. It is prudent for the hospital to extend the implementation of its antibiotic stewardship program beyond inpatients to include outpatients. This should include developing antibiotic prescribing guidelines based on established evidence to guide appropriate prescribing in the OPD. 

(b) *AWaRe* antibiotics: Antibiotics belonging to the *Watch* group were prescribed in almost half of the antibiotic prescriptions every month. *Watch* antibiotic use in this study is higher than that found in Finland (23%) but lower than in Iran (77%) [15]. Antibiotics in the *Watch* group are at higher risk of developing AMR compared to *Access* antibiotics. Reserve antibiotic use was, however, low across the year, which is encouraging. To optimize prescribing, an antibiotic treatment formulary focused around the WHO *AWaRe* categories is needed to increase *Access* group use, reduce *Watch* antibiotics, and keep *Reserve* use low. Routine review meetings at the hospital to monitor the appropriateness of antibiotic usage based on clinical conditions of the patients will help ensure guideline compliance.

(c) Completeness of antibiotic prescriptions: We observed that almost all of the prescriptions had adhered to good documentation, which includes mentioning the drug, dose, duration, route, frequency, and diagnosis. The hospital EMR system included a module for prescribing, which ensured efficient documentation. The availability of this system is a valuable resource that will facilitate future audits. There is scope for further strengthening of this mechanism wherein a few of the variables must be entered in a standard format and not as open-ended fields. For instance, the diagnosis did not follow any standard disease classification, and therefore could not be included in the analyses. Using WHO’s International Classification of Diseases (ICD) [43,44] could be one way of standardizing this parameter in the EMR and thus allow for global comparisons. 

(d) Types of antibiotic prescription: The common types of antibiotics prescribed included amoxicillin–clavulanic acid, azithromycin, ciprofloxacin, metronidazole, and cefuroxime. The most prescribed antibiotics among the *Access* group were amoxicillin–clavulanic acid, metronidazole, and tetracycline, while for the *Watch* group azithromycin, ciprofloxacin, and cefuroxime were the most prescribed. Similar results were reported from other studies from Ghana and Vietnam [28,45]. Going forward, it would be desirable to develop antibiograms specific to the OPD to ensure prescribed antibiotics are appropriate. This would require increased culture and drug susceptibility testing in addition to enhancing the capacity and efficiency of the microbiology department in the hospital.

The study has the following strengths. First, is the large dataset from the EMR which was available and used for analyses. Second, the completeness of study variables opted for this study was up to 98%. Third, the study was conducted and reported in accordance with the Strengthening the Reporting of Observational Studies in Epidemiology (STROBE) guidelines statement [46]. There are few limitations of the study. The data was from only one hospital, which may limit the generalizability of the study findings. Additionally, the format in which data is recorded in the EMR for certain important parameters, such as diagnoses, precluded analyses such as appropriateness of antibiotic prescriptions. For the same reason, we were not able to conduct statistical analysis to assess the factors influencing prescription practices.

## 5. Conclusions

There was high antibiotic usage in the hospital when compared to WHO standards. The use of antibiotics from the *Watch* group was widespread. The antibiotic prescription parameters were well-documented. Extending antimicrobial stewardship to the OPD settings by developing standard guidelines, an OPD specific drug formulary and antibiograms can promote rational antibiotic use and improve patient outcomes in the hospital.

## Figures and Tables

**Table 1:** Demographic and clinical characteristics of patients attending the OPD of University Hospital, KNUST, between January and December 2021.

Characteristics		Number	(%)
Total number of patients		49,660	
**Demographic characteristics**			
Age in years ^1^			
	0–14	6301	(12.7)
	15–24	22,705	(45.7)
	25–34	7501	(15.1)
	35–44	4004	(8.1)
	45–54	3312	(6.7)
	55–64	2867	(5.8)
	≥65	2964	(5.9)
Sex			
	Male	26,783	(53.9)
	Female	22,877	(46.1)
**Clinical characteristics**			
Chronic conditions ^2^	Any chronic condition recorded	5242	
	Hypertension	3584	(68.4)
	Diabetes	1059	(20.2)
	Asthma	599	(11.4)
**Clinic**			
	General OPD	39,186	(78.9)
	Dental	2108	(42.0)
	Diabetes/Hypertension clinic	517	(1.0)
	Others	7849	(15.8)
**Patient encounters**			
	1	6108	(12.3)
	2	7263	(14.6)
	3	10,542	(21.2)
	4	9827	(19.8)
	5	7516	(15.1)
	6	4287	(8.6)
	>6	4117	(8.3)

^1^ Six implausible age values were dropped for this variable. ^2^ Patients were counted more than once if multiple categories applied, percentages calculated from patients with recorded chronic medical conditions; KNUST—Kwame Nkrumah University of Science and Technology; *AWaRe*—Access, Watch, Reserve; OPD—outpatient department.

**Table 2:** Patient encounter characteristics associated with antibiotic prescriptions in the outpatient department of University Hospital, KNUST, in Ghana between January and December 2021.

Characteristic	Total Patient Encounters	Antibiotic Prescribed ^1^	
	N	N	(%)
**Age groups (in years)**			
0–14	11,198	6103	(54.5)
15–59	82,219	29,759	(36.2)
≥60	16,857	3473	(20.6)
**Sex**			
Male	47,271	17,241	(36.5)
Female	63,009	22,098	(35.1)
**Clinic**			
General OPD	81,243	30,089	(37.0)
Dental	2705	103	(3.8)
Diabetes/Hypertension clinic	5050	2749	(54.4)
Others	21,282	6398	(30.1)

KNUST—Kwame Nkrumah University of Science and Technology. ^1^ Percentages calculated with total number of individuals who received any antibiotic as denominator.

**Table 3:** Pattern of antibiotic prescriptions in the outpatient department of University Hospital, KNUST, in Ghana between January and December 2021.

Antibiotic	Anatomic Therapeutic Classification	Number of Prescriptions	(%) ^1^
**Access group (N = 28,152)**			
Amoxicillin–Clavulanic Acid	Beta-lactam/beta-lactamase-inhibitor	13,944	(49.7)
Metronidazole	Imidazoles	5956	(21.2)
Tetracycline	Tetracyclines	1968	(7.0)
Doxycycline	Tetracyclines	1316	(4.7)
Clindamycin	Lincosamides	1060	(3.8)
Flucloxacillin	Penicillins	953	(3.4)
Secnidazole	Imidazoles	725	(2.6)
Others ^2^		432	(1.2)
**Watch group (N = 27,395)**			
Azithromycin	Macrolides	7995	(29.2)
Ciprofloxacin	Fluoroquinolones	7712	(28.1)
Cefuroxime	Second-generation-cephalosporins	5549	(20.2)
Neomycin	Aminoglycosides	2097	(7.6)
Tobramycin	Aminoglycosides	1678	(6.1)
Levofloxacin	Fluoroquinolones	682	(2.5)
Ceftriaxone	Third-generation-cephalosporins	490	(1.8)
Ofloxacin	Fluoroquinolones	381	(1.4)
Clarithromycin	Macrolides	365	(1.3)
Cefixime	Third-generation-cephalosporins	287	(1.0)
Others ^3^		109	(0.5)
**Reserve group (N = 1045)**			
Polymixin B	Polymyxins	1045	(100)
**Not recommended (N = 1798)**			
Ciprofloxacin–Tinidazole combination	-	1798	(100)
**Unclassified (N = 451)**			
Bacitracin	Polypeptide antibiotic	263	(58.3)
Mupirocin	-	188	(41.6)

^1^ Percentage calculated with total number of prescriptions of an antibiotic from corresponding *AWaRe* category as denominator. ^2^ Other antibiotics belonging to *Access* group (amikacin, gentamicin, chloramphenicol, nitrofurantoin, benzylpenicillin, cloxacillin, penicillin and cotrimoxazole), ^3^ Other antibiotics belonging to *Watch* group (vancomycin, fusidic acid, meropenem, cefaclor, fosfomycin, cefpodoxime, and erythromycin), KNUST—Kwame Nkrumah University of Science and Technology.

## Data Availability

Requests to access these data should be sent to the corresponding author.

## Open Access Statement and Disclaimer

In accordance with WHO’s open-access publication policy for all work funded by WHO or authored/co-authored by WHO staff members, WHO retains the copyright of this publication through a Creative Commons Attribution IGO license (http://creativecommons.org/licenses/by/3.0/igo/legalcode accessed on 12 July 2022) which permits unrestricted use, distribution, and reproduction in any medium provided the original work is properly cited. There should be no suggestion that WHO endorses any specific organization, products, or services. The views expressed in this article are those of the authors and do not necessarily reflect those of their affiliated institutions. The use of the WHO logo is not permitted. This notice should be preserved along with the article’s original URL.
